# Factors influencing the high rejection rates of HIV 1/2 serology samples at Charlotte Maxeke Johannesburg Academic Hospital and the cost implications

**DOI:** 10.4102/sajhivmed.v23i1.1326

**Published:** 2022-01-11

**Authors:** Bhaveshan Reddy, Naseem Cassim, Florette Treurnicht, Zinhle Makatini

**Affiliations:** 1 Department of Virology, Faculty of Health Science, University of the Witwatersrand, Johannesburg, South Africa; 2 National Health Laboratory Service, Johannesburg, South Africa; 3 Department of Haematology and Molecular Medicine, Faculty of Health Sciences, University of the Witwatersrand, Johannesburg, South Africa

**Keywords:** HIV, rejection rates, cost analysis, laboratory, diagnostics

## Abstract

**Background:**

HIV enzyme-linked immunosorbent assay (ELISA) is one of the most requested test sets within Virology and forms an essential part of patient management. Assessment of the rejection criteria is a key quality indicator, crucial for improving laboratory services and efficiency to ensure accurate and reliable results.

**Objectives:**

The aim of this study was to identify the factors that influence the HIV 1/2 serology rejection rates (RR) at Charlotte Maxeke Johannesburg Academic Hospital and to evaluate the associated costs.

**Methods:**

A retrospective study was conducted (June to December 2019) to identify the RR and rejection criteria of HIV serology samples throughout the total testing process. Descriptive analysis using percentages and frequencies was used to analyse the RR by phase, health establishment, ward and healthcare professional. A cost analysis incorporating minor and major costs was modelled in each phase of testing, and the total cost of rejections was calculated.

**Results:**

A total of 6678 tests were received, and 738 were rejected (RR = 11.1%). The pre-analytical phase contributed significantly to the overall RR, with the requirement of a separate sample (57.44%) the most common reason for rejection. The total cost per rejected test was $2.47, which amounted to a total rejection cost of $197.55, of which $158.18 was caused by the pre-analytical rejection criteria.

**Conclusion:**

High RR of HIV tests were noted, resulting in significant cost wastage. Identification and analysis of rejections must be implemented across all laboratories to improve the efficiency of testing, provide a cost-saving benefit and maintain high laboratory standards.

## Introduction

HIV remains a leading cause of increased morbidity and mortality, especially in Southern Africa. Despite active measures to control the course of this disease, over 7 million individuals are living with HIV in South Africa.^1^ The Joint United Nations Programme on HIV/AIDS (UNAIDS) 95-95-95 goals highlight the role of diagnostic testing as one of the main strategies in controlling this pandemic.^2^ Providing accurate and reliable results timeously has proven to have positive outcomes in the management of HIV-infected individuals.^3^ In line with the 95-95-95 targets, South Africa adheres to the universal test and treat strategy. As such, the laboratory has a responsibility to provide quality results to promote patient safety. A constant increase in test demand results in increased workload, which leads to inefficiencies within the laboratory and healthcare facility. As a result, laboratory accountability for patient safety has been highlighted in recent studies and should include the monitoring and analysis of key quality indicators such as rejection rates (RR).^4^ This can be tailor-made to accommodate different laboratories; however, when doing so, pre-existing limitations such as laboratory design, infrastructure, personnel and operating processes must be considered.

The rejection of a sample has detrimental consequences for the laboratory, health facility and individual tested. This can be reflected in delayed turnaround times, reduced efficiency, poor workflow, cost implications, missed or delayed diagnostic opportunities and loss to follow-up.^5,6^ A rejected test caused by laboratory error or an HIV test that is incorrectly requested in a setting such as the early infant diagnosis (EID) programme can be a major pitfall in achieving targets that aim to reduce new paediatric infection rates (0–24 months). Several studies reported that a significant number of rejected tests are not repeated (once-off occurrence) and can account for up to a 12% increased chance of inappropriate patient care.^7^ A projected error rate of this magnitude in the South African context can apply tremendous pressure to the already financially constrained and resource-burdened public health sector, especially in key age categories such as the paediatric population. Recent literature reports several factors contributing to increased RR throughout the total testing process, including haemolysis, the mislabelling of samples and inappropriate sample collection.^8^ The total testing process consists of three phases – (1) pre-analytical, (2) analytical and (3) post-analytical – with the majority of rejections occurring in the pre-analytical phase.^9^

With the rise in the burden of diseases and the emergence of novel pathogens, there has been an increased need for diagnostic testing; however, this need has not been met with the necessary financial resources. To support the increase in testing, there is a need to review cost-saving strategies and how they will benefit the laboratory as well as the patient. A costing analysis that used the Markov probability model suggested a total loss of $357.15 per hospital patient.^10^ Similarly, a number of studies have estimated pre-analytical RR costs to range between $160.00 and $225.00 per month.^10,11^ These substantial costs can have a significant impact on the total hospital budget.

The primary aim of this study was to assess the HIV serology RR and reasons in the Department of Virology, National Health Laboratory Service (NHLS), Charlotte Maxeke Johannesburg Academic Hospital (CMJAH). The secondary aim was to undertake a costing analysis to illustrate the financial implications of these rejections in a typical South African health laboratory.

## Materials and methods

### Study setting

This retrospective study was conducted for the period 01 June 2019 to 31 December 2019 in the Department of Virology of the NHLS based at CMJAH (Johannesburg, South Africa). The department provides a dedicated 24-h diagnostic service to CMJAH, a tertiary-level hospital, surrounding primary healthcare (PHC) facilities and both regional and district level hospitals.

### Specimen registration

For HIV serology test requests, samples were registered on the laboratory information system (LIS) following standard operating procedures to capture all demographic, clinical and test request details provided on the laboratory request form. The test results are automatically downloaded to the LIS, and all laboratory data are stored in the NHLS corporate data warehouse (CDW).

### Test rejections and rejection criteria

All rejection codes and descriptions were defined by the local laboratory and the NHLS expert committees. Electronic gatekeeping of samples was included as a rejection criterion in this study, defined as any HIV serology sample requested before the minimum request interval time had elapsed. The pre-analytical phase included all processes from sample collection to receipt at the CMJAH NHLS receiving office. All processes that pertained to the performance of the test were assigned to the analytical phase, whereas the post-analytical phase involved the analysis, interpretation and authorisation of the test results.

### Lookup table

For each rejection, a rejection code is entered into the LIS to indicate to the requesting healthcare practitioner the reason for not performing the test, for example ‘specimen insufficient’ (SPINS). Because of the vast number of rejection codes reported, a lookup table was developed using Microsoft Excel (Redmond, California, United States) to assign the rejection status (rejected/not rejected) and phase of testing (pre-analytical, analytical or post-analytical). Lookup tables were then used to group assigned codes of large data sets with similar information, avoiding manual coding (Table 1).

**TABLE 1:** Example of some rejection codes and rejection reasons used to assign the rejection status and rejection phase values in a lookup table for HIV serology samples.

Rejection code	Rejection reason description	Rejection status	Rejection phase
RSEP	Require separate specimen	Rejected	Pre-analytical
SPINS	Specimen insufficient	Rejected	Pre-analytical
NDLE	Not done: lab error	Rejected	Analytical
ONCOR	Not done: non-reportable result	Rejected	Post-analytical
CEGK	Electronic gatekeeping	Rejected	Pre-analytical

### Data analysis

An NHLS-specific test code for HIV serology was used to extract data from the CDW for the 6 months (01 June to 31 December 2019). All rejected tests and the reasons for rejection were captured on the LIS and downloaded to the CDW database. The data extract also included the following variables: (1) age, (2) facility name, (3) referring healthcare professional, (4) rejection code, (5) rejection reason description, (6) date of collection, (7) date of rejection, (8) ward code and (9) ward description. Age was stratified into three categories – infants and toddlers (0–24 months), children and adolescents (2–18 years) and adults (> 18 years) – and the total number of rejections per age group was calculated. We used the facility name to assign the health establishment type (PHC facility or hospital). Similarly, we used ward descriptions to assign the following ward types: (1) medical, (2) trauma and casualty, (3) surgical, (4) intensive care unit (ICU), (5) paediatrics, (6) obstetrics and gynaecology and (7) antiretroviral (ARV) clinic. Experts read the ward description and assigned the ward type; for example ‘Area 165 Medical Casualty CAS’ was assigned as the trauma and casualty ward.

Requisitioner information, which included the healthcare practitioner’s name and professional society registration details (South African Nursing Council [SANC] or Health Professions Council of South Africa [HPCSA]), was used to assign the following professional types: (1) nurse, (2) medical intern (IN) and (3) medical practitioner (MP). For example, requisitioner details that included SANC, IN and MP numbers were assigned as a nurse, intern and medical practitioner, respectively. Data that did not include the rejection reason description could not be categorised and were excluded from this study.

### Rejection rate calculations

The RR were calculated using the formula:


$$\left(\text{RR}=\frac{\text{Rejections}}{\text{Total test volume}}\times 100\right)$$ [Eqn 1]

and reported as a percentage. The RR were analysed by process phase, health establishment, ward and healthcare professional.

### Cost analysis

The cost analysis was performed to determine the cost per rejection for each phase of testing. All costs were obtained in South African rands (ZAR) and converted to United States dollars (USD) using an exchange rate of 14.60/$1.00. The accounting stance was assumed to be the provider of diagnostic services, and costs associated with overheads and laboratory management were excluded. For the pre-analytical phase, data generated from a local study that conducted a top–down costing of historical expenditure data for the 2019–2020 financial period for the CMJAH receiving office were used ($0.77 per registration). A pre-analytical cost per test was calculated using the assumption that on average 3.5 tests are requested per registration.

For the analytical phase (pre-analytical cost + analytical cost), the cost per test associated with the analyser, staffing, reagents and test consumables was determined. These costs were obtained from the Oracle Enterprise Resource Planning (ERP) system of the NHLS (Box 1). The Roche Cobas 8000 modular analyser (module e602; Roche Diagnostics, Basel, Switzerland) was provided through a service placement agreement, and thus the costs associated with the outright purchase, maintenance and servicing of the instrument are included in the reagent cost. All costing data were captured in Microsoft Excel for analysis.

BOX 1 Itemised list of resources within the analytical phase of testing.

**Itemized list of resources**

HIV combi reagent and calibrator

HIV control

Clean cell buffer M 2 × 2 L

ProCell M

Probe clean M

Probe wash M

Assay tip/cup and waste box

Medical technologist (C2) (5 min)

For staffing costs (medical technologist – C2 grade), given the short sample preparation time, the time to perform the test (in minutes) was multiplied by the annual cost per minute, based on the assumption that HIV serology testing is offered on a 365-day running cycle (annual cost / 365). Similarly, for the post-analytical phase, the average time (3 min) for a registrar (D1 grade) to review and authorise the result on the LIS was used. All staff salaries were based on the NHLS cost to company (CTC) salary scales.

To calculate the total cost of rejection, the cost per test was multiplied by the total number of HIV serology rejections. Corporate warehouse data were provided as a CSV file used for preliminary analysis in Microsoft Excel. Descriptive analysis was conducted using TIBCO Statistica version 13.5.0 analytical software (California, United States).

### Ethical considerations

The study was approved by the Human Research Ethics Committee, University of the Witwatersrand (clearance certificate number M201117).

## Results

**FIGURE 1:**
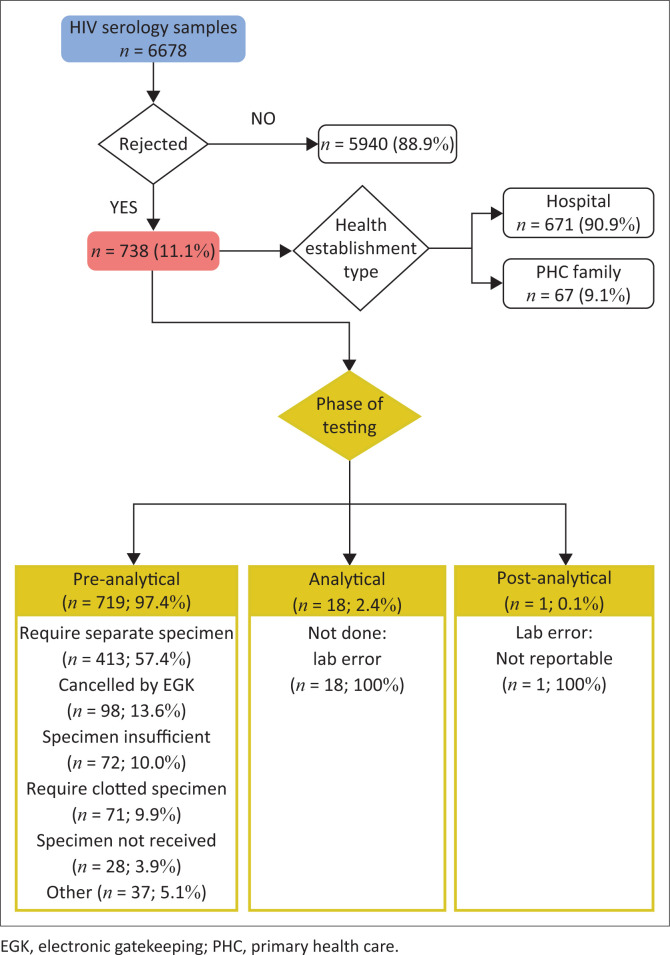
Rejection rates and criteria throughout the total testing process for HIV serology samples performed at the Department of Virology, National Health Laboratory Service, Charlotte Maxeke Johannesburg Academic Hospital, South Africa, between 01 June 2019 and 31 December 2019.

A total of 6678 HIV serology samples were received for the study period, of which 738 samples were rejected (11.1%). Among the rejected group, a mean age of 32 years was reported (standard deviation: 20.57), with the majority attributed to the adult population (560/738; 75.88%). Within the age group of 0–24 months, there were 106 rejected samples (14.36%). There were 671 (90.92%) and 67 (9.08%) rejected samples from hospitals and PHC facilities, respectively (Figure 1).

The majority (719/738; 97.4%) of rejections occurred in the pre-analytical phase, where most samples (413/719; 57.4%) were rejected because of the requirement for a separate sample (Figure 1). This was followed by rejections as a result of electronic gatekeeping (13.6%), SPINS (10%), requiring a clotted sample (9.9%) and specimen not received (3.9%). Other (5.1%) reasons for sample rejection in the pre-analytical phase included mislabelling, incomplete healthcare worker or patient information, and unsuitable samples. Eighteen (2.43%) samples were rejected in the analytical phase because of a number of laboratory errors, such as poor sample integrity, and one (0.13%) in the post-analytical phase because of a non-reportable result.

**FIGURE 2:**
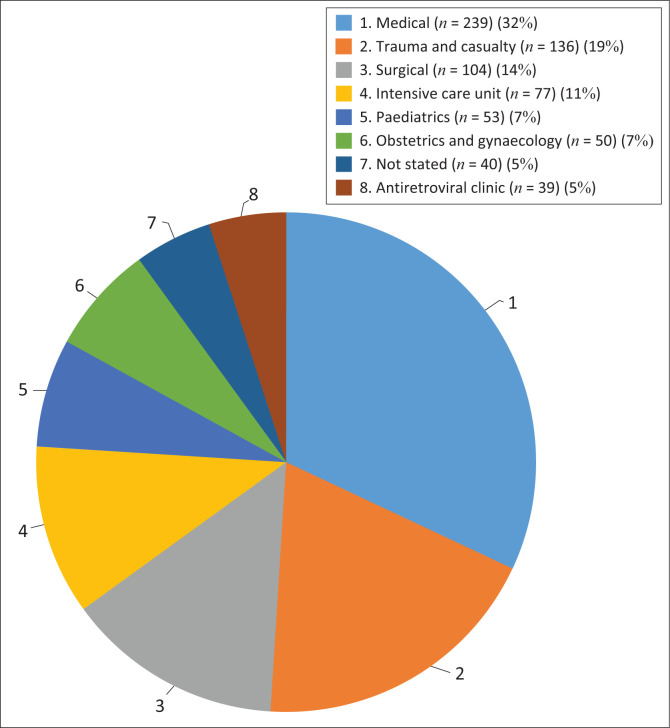
Pie chart displaying the percentage of HIV serology sample rejections by ward type at the Department of Virology, Charlotte Maxeke Johannesburg Academic Hospital, South Africa between 01 June 2019 and 31 December 2019. The intensive care unit metric includes both the adult and paediatric age categories.

Based on the ward type, the medical unit had the highest number of rejections (239/738; 32%) followed by trauma and casualty (136/738; 19%), surgical (104/738; 14%) and ICU, which accounted for 11% (77/738) (Figure 2). The requirement of a separate sample was the most common reason for rejection across all departments, health facilities and age groups. Within this criteria, hospital samples accounted for 375/413 (90.80%) and clinics for 38/413 (9.20%) rejections. Transport of samples was a rejection criterion associated with clinic samples only (3/67; 4.47%).

Further analysis by health establishment and healthcare profession type revealed that, at hospitals, the majority of rejections were requested by medical practitioners (543/671; 80.92%). In contrast, interns, nurses and clinical associates accounted for the remaining 19%. For PHC facilities, 79.1% of rejections were requested or collected by nursing staff (Table 2).

**TABLE 2:** Number of HIV serology samples rejected, by health establishment and healthcare professional type.

Health establishment type	Healthcare professional type	Number of rejected samples (*N* =738)	
		*n*	%
Hospital	Medical practitioner†	543	80.92
	Intern	100	14.90
	Nurse	16	2.38
	Clinical associate	12	1.80
Total	-	671	100.00
Primary health care facility	Nurse	53	79.10
	Medical practitioner	11	16.41
	Intern	3	4.49
Total	-	67	100.00

Note: Data are reported for the Virology Department at the Charlotte Maxeke Johannesburg Academic Hospital, South Africa, between 01 June 2019 and 31 December 2019.

† , ‘Medical practitioner’ includes community service officer, medical officer, registrar and consultant.

Table 3 summarises the itemised costs for HIV serology by testing phase. The total cost per sample for the pre-analytical phase was calculated to be $0.22. The bulk of the pre-analytical costs was for staff, at $0.13 per sample. For the analytical phase, a cost per test of $1.83 was reported. The HIV reagent pack contributed $1.04, compared to $0.50 for buffer and waste consumables. The staff cost contributions for the analytical and post-analytical phases were $0.29 and $0.42, respectively (Table 3).

**TABLE 3:** Itemised cost per sample for each phase of testing for HIV serology rejections.

Item in each phase of testing	Cost per sample (USD)
**Pre-analytical**	**0.22**
Collection and registration	0.05
Laboratory equipment	0.01
Staff	0.13
Operating costs	0.03
**Analytical**	**1.83**
HIV reagent pack	1.04
Buffer	0.26
Waste consumables	0.24
Staff	0.29
**Post-analytical**	**0.42**
Staff	0.42

USD, United States dollars.

The total cost per sample was calculated to be $2.47 across the three phases of testing. The total cost of rejections was $197.55 (Table 4). The pre-analytical phase contributed 82.6% of the total cost of rejections ($158.18), followed by the analytical phase, with 17.2% ($36.90). Given the single post-analytical rejection, the total cost for that phase of testing was $2.47.

**TABLE 4:** Determining the total cost of rejections for HIV serology testing across the three phases of testing.

Phase of testing	Cost of rejection per sample (USD)	Number of rejections		Total cost of rejections (USD)
		*n*	%	
Pre-analytical	0.22	719	82.6	158.18
Analytical	2.05	18	17.2	36.90
Post analytical	2.47	1	0.2	2.47

**Total**	**2.47** †	**738**	**100.0**	**197.55**

USD, United States dollars.

† , The total cost is calculated incorporating the itemized cost in all three phases of the total testing process (based on a single rejected sample).

## Discussion

In this study, we assessed the rates, reasons and cost of rejections for HIV serology tests at the Department of Virology at an academic hospital in South Africa over 6 months in 2019. Overall RR of 3.6% were reported for all test requests received during 2019 (CMJAH NHLS statistics, unpublished). This is consistent with other studies that reported similar average RR (0.1% – 3.49%).^12,13^ However, the HIV serology RR of 11.1% reported in this study are significantly higher than reported rates and not aligned with the accepted internal test RR of < 5% set for Virology.

In the laboratory setting described, multiple reasons for rejection throughout the total testing process were noted. As reported in other studies, the pre-analytical phase was identified as the main phase of rejection, where 1 in 10 patients had a missed or delayed diagnosis, predominantly as a result of the requirement for a separate sample. This criterion is consistently noted as the most common reason for rejection in the data described here and highlights a gap in training on HIV serology sample collection for healthcare practitioners.

In addition, despite current EID guidelines, which recommend that an HIV DNA polymerase chain reaction (PCR) test be done from birth to 18 months of age, 14% of rejected tests fell within this age group. Our data identified that HIV serology tests are incorrectly requested within this age group, indicating a need for clinical training on guidelines and laboratory requirements for testing. It further underlines the importance of routine monitoring and analysis of the rejection criteria as a key quality indicator for continuous improvement.

This study also illustrates the laboratory’s acceptability criteria and how strictly they are adhered to. Within the receiving laboratory, all staff are guided by the standard operating procedures for registration of a sample and the criteria for rejection. A large number of rejection criteria also fall within the clinical domain, over which the laboratory may not necessarily have influence and which requires tighter control with regard to the collection and requisition of samples by clinical departments. Other studies have shown similar findings, in that a large number of errors (pre-analytical) occur outside the laboratory and are caused by actions predominantly by the healthcare workers.^14^ This is attributable to frequent staff rotation and substandard training. It is clear from the data of this study which healthcare personnel requested or drew samples that led to rejections and would benefit from additional training. All these aspects are clearly described in the NHLS handbook provided to hospitals and clinics. As such, these errors could have easily been avoided, subsequently eliminating a large percentage of rejections. It is important to note that the rejected tests originate mainly from the medical, trauma and casualty, and surgical wards, where timeous and accurate HIV results are important for clinical management of patients because of the high burden of HIV in South Africa.

In response to these findings, despite the lack of standardised rejection criteria amongst laboratory networks, appropriate corrective and preventative actions can be implemented, monitored and assessed. These include regular staff training and routine competency assessments directed towards key receiving office staff and healthcare workers. These training mechanisms will provide the greatest outcome in terms of RR reduction but should also be extended to all healthcare and laboratory workers. Laboratory manuals targeting key issues such as requesting a separate sample for HIV testing can avoid delays in turnaround time and patient management. Training on the current HIV and EID guidelines, as well as providing itemised rejections for a specific test set by ward, may also aid in the regular monitoring and evaluation of procedures, which will ensure good diagnostic practices.

Reduced RR may lead to improved patient care.^15^ The impact of rejecting samples also has financial implications. The current study findings assessed the cost implications of these rejections. For one laboratory, an annual cost of $383.03 was reported.^16^ When these data are extrapolated for national HIV serology testing across the NHLS for the 2019–2020 financial period (assuming 11.1% RR), the total cost of rejections amounts to $122 295. This signifies a substantial cost burden to the health system that could be avoided. Furthermore, the cost of rejections must be seen as a contributing loss within the broader healthcare system. Improving the efficiency of testing will have a twofold benefit, firstly providing a significant contribution to healthcare resource savings and secondly improving a key quality indicator that plays a pivotal role in maintaining high laboratory standards.^17,18^ As the rejections occurred primarily within the pre-analytical phase in this study, it affirms that measures put in place through detailed rejection criteria by the NHLS also served to reduce the costs associated with performing unnecessary test or tests with compromised quality through the use of shared samples.^19^

The study limitations included a small number of unspecified rejection reasons from the data extract, as well as the rejection reasons being grouped into similar categories. This may have resulted in non-specific rejection codes being used that therefore did not necessarily accurately describe the rejection. The data from this study only take into consideration HIV laboratory-based testing and exclude a large volume of point-of-care testing performed at other health establishments. However, because of the availability of such data, this study recognises an opportunity to improve the analysis of RR if the data are managed properly and categorised in a more user-friendly manner. This will allow for laboratories to monitor their data over shorter time intervals and will lead to the timely identification of problem areas. This will encourage proactive measures to be implemented in order to reduce the overall number of rejections.

## Conclusion

There are substantial data to suggest that inappropriate test use, rejections and repeat testing contribute to increased laboratory expenditure and unfavourable patient outcomes. Identification of the key RR is an additional tool to monitor laboratory efficiency, improve service delivery and identify areas in the testing process that need intervention through corrective actions. Limiting rejections in the laboratory will save significant costs and time and improve the clinical utility of diagnostic tests, which will benefit the laboratory, patients and the healthcare system.
